# Odontogenic tumors in Ethiopia: eight years retrospective study

**DOI:** 10.1186/s12903-017-0347-8

**Published:** 2017-02-16

**Authors:** Bruktawit Kebede, Dawit Tare, Birke Bogale, Fessahaye Alemseged

**Affiliations:** 1 Oral and Maxillofacial Surgery case team, Department of Dentistry, Jimma University, Jimma, Ethiopia; 2 Department of Surgery, Jimma University, Jimma, Ethiopia; 3 Department of Dentistry, Millennium Medical College, St. Paul’s Referral Hospital, Addis Ababa, Ethiopia; 4 Department of Epidemiology, Jimma University, Jimma, Ethiopia

**Keywords:** Complication, Ethiopia, Pattern, Surgical treatment, Odontogenic tumors

## Abstract

**Background:**

Odontogenic tumor (OT) comprises a large heterogeneous group of lesions arising from tooth producing tissues or its remnants. Studies on OTs are scarce in Ethiopia. Thus, the present study aimed to assess the pattern of OTs in Ethiopia.

**Methods:**

An 8 years retrospective study was conducted at the Dental and Maxillofacial Department, St. Paul’s referral hospital, Addis Ababa, Ethiopia. Data were collected by reviewing the medical records of patients who visited the Department from September 2008 to August 2015. All the collected data were coded, checked, edited and entered to SPSS windows 18. Lastly, descriptive statistics, and logistic regression were performed for data analysis.

**Results:**

A total of 448 patient’s socio-demographic, and clinical data were reviewed from the registry book of patients diagnosed with OT. Of these, only 163 patient’s records were complete and suitable for the study. 88 (54%) of the study subjects (163) were males, while the remaining 75 (46%) were females. The mean age of patients was 34, ranging between 8 and 80 years. 132 (81.0%) of the OTs were benign, and the rest 31 (19.0%) were malignant type. 126 (77.3%) of OTs occurred in the mandible, and the remaining 37 (22.3%) were located in the maxilla. 135 (82.8%) of the patients had primary surgical treatment. Continuity defect, facial disfigurement and malocclusion were the most frequently encountered complications after surgery. Living in rural areas showed statistically significant association with complication after surgery [Adjusted OR = 2.13, (95% CI: 0.98, 4.6)]. In addition, tumor size had statistically significant association with complication after surgery [Adjusted OR = 4.24, (95% CI: 1.76, 10.21)].

**Conclusion:**

OTs were mainly found in males than their females counterpart. Benign OT was predominant over malignant OTs. Regular checkup and/or visit to dentists could help early case detection, and management of OTs.

## Background

Odontogenic tumors (OTs) are rare tumors that are specifically seated in the jaw bones. They constitute a heterogeneous group of lesions due to the different degrees of intertissue interaction and various growth patterns. OTs are derived from epithelial, ectomesenchymal and/or mesenchymal elements of the odontogenic tissues. OTs show variable clinical and histopathological features. Among all, the ability of OTs to transit from one form to another complicates the formal classification of OTs. As a result, World Health Organization (WHO) was obligated to revise its 1971 classification of OTs in 1992 to have consensus all over the world [1, 2]. Nevertheless, this revision could not resolve the controversial classification of OTs. In 2005, WHO published the latest third edition of OTs histological typing that brought non-negligible consensus. Of note, the WHO classification has divided OTs as epithelial, mesenchymal, and mixed based on the tissue they originated [3–6].

Many retrospective studies have been conducted in different continents of the world such as Africa, Asia, Europe, and North and South America to assess the distribution of OTs. These studies reported different distribution rates of OTs that ranged from 1 to 28%. The overall and relative frequency of individual OTs differ from region to region. It is speculated that the differences in the observed frequencies are attributed to variations in geographic or cultural settings [7]. Importantly, most forms of tumors are age related. In Ethiopia, the average life expectancy at birth in 2014 was 64.1 years [8].

The cause(s) of OTs remain(s) unclear. Nonetheless, the majority of OTs seem to arise *de novo*, without an apparent causative factor [9–11]. It has been reported that OTs have a predilection for the entire facial region specifically, for the mandible and maxilla [12]. The treatment of choice for OT is surgical operation; extirpation and curettage for benign type, and segmental resection for malignant type of OT. If left untreated, it could result in death within four to six months of diagnosis [12]. Despite these consequences, little is known about the magnitude of OT in Ethiopia. Therefore, this study determined pattern of odontogenic tumors in Ethiopian population.

## Methods

This study received ethical approval from Jimma University, College of Public Health and Medical Sciences Ethical and Review office. Patient confidentiality was strictly maintained by not mentioning patient’s name and any potential identifier on the checklist.

A retrospective cross-sectional study design was conducted to determine the pattern of OTs in Ethiopia at Dental and Maxillofacial Department, St. Paul’s referral hospital, Addis Ababa, Ethiopia. The hospital has a catchment population of more than 5 million (https://en.wikipedia.org/wiki/St._Paul%27s_Hospital_Millennium_Medical_College). This Department is one of the largest and pioneer centers for maxillofacial surgery in Ethiopia. The vast majority of the cases investigated at this Department are patients who are referred from all over the country. To this end, we reviewed the medical recordings of patients who were diagnosed as OT from September 2008 to August 2015 at the Department.

Complete medical records of patients with unambiguous histopathological diagnosis of OT were included in this study. Importantly, histopathological examinations were done by senior pathologists for the purpose of patient diagnosis. All the necessary data such as age, gender, present address, duration of OT lesion, location of the OT lesion, size of OT lesion, histopathology findings, type of operation and complication after surgery were collected by well oriented nurses working at the Department. The completeness and quality of all collected data were assessed at the end of each data collection dates by the principal investigator.

Lastly, descriptive statistics and logistic regression analysis were performed using Statistical Package for Social Sciences (SPSS) version 18. Odds ratios were used as measures of association between the independent and outcome variables. A p-value of less than or equal to 0.05 was considered statistically significant.

## Results

### Recruitments

**Fig. 1:**
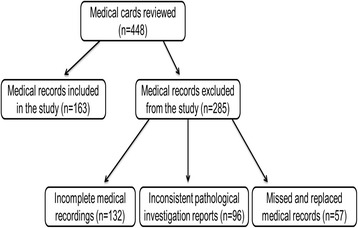
Flow diagram of recruitments. Medical records of patients who were diagnosed as OT from September 2008 to August 2015 were reviewed

A total of 448 patient’s medical records with the diagnosis of OT were reviewed. Of these, 285 recording were excluded from this study due to various reasons. First, 132 (29.5%) medical records had an incomplete medical recording. Second, 57 (12.7%) records were missed and replaced with new records upon the consecutive follow-up appointments. Third, 96 (21.4%) records revealed inconsistent histopathological investigation reports of biopsy and fine needle aspiration cytology upon repeated examinations. As such, only 163 (36.4%) patients’ medical records were complete and found to be suitable for the study (Fig. 1).

### Socio-demographic characteristics

From the total of 163 reviewed medical records, 88 (54%) were males, while the remaining 75 (46%) were females. The majority, 82 (50.3%), of patients were within age group of 20–39 years. 96 (58.9%) of the participants were living in rural areas of the country (Table 1).

**Table 1:** Socio-demographic characteristics of patients with the diagnosis of OT at St. Paul’s referral hospital, Addis Ababa, Ethiopia

Socio-demographic characteristics	Frequency (%)
Gender	
Male	88 (54)
Female	75 (46)
Age	
0–19	34 (20.8)
20–39	82 (50.3)
> = 40	47 (28.8)
Address	
Urban	67 (41.1)
Rural	96 (58.9)

### Pattern of odontogenic tumors

**Fig. 2:**
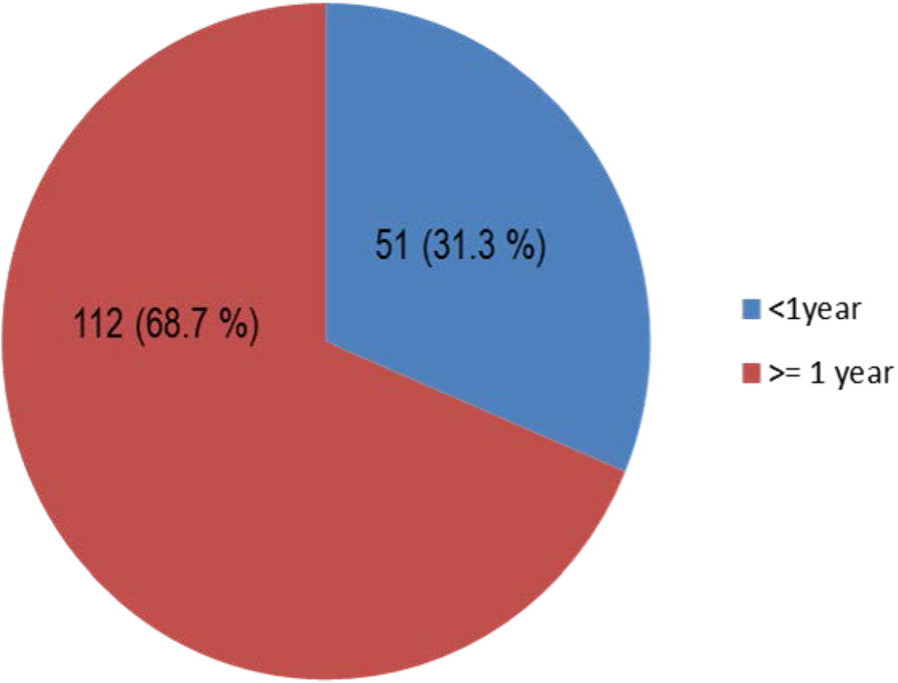
Presentation of odontogenic tumors. OT cases presented less than or greater than 1 year of onset of symptoms were presented

Of the 163 cases, 80.4% were benign tumors, while the remaining 19.6%, were malignant tumors. Ameloblastoma 75 (46%) was the most frequent type of benign OT, followed by odontogenic myxioma (8.6%). On the other hand, primary intraosseous squamous cell carcinoma (IOSCC) was the most common type of malignant OT (Table 2). The majority, 112 (68.7%), of OT cases were presented after 1 year of onset of symptoms. On the other hand, 51 (31.3%) of the cases were presented before 1 year of onset of symptoms (Fig. 2).

**Table 2:** Frequency distribution of odontogenic tumors at St. Paul’s referral hospital, Addis Ababa, Ethiopia

Types of odontogenic tumor		Frequency (%)	Frequency (%)
Being tumors	Ameloblastoma	75 (46.0)	131 (80.4%)
	Odontogenic myxioma	14 (8.6)	
	Keratocystic OT	10 (6.1)	
	Odontogenic fibroma	10 (6.1)	
	Ameloblatic fibroma	9 (4.3)	
	Odontoma	6 (3.7)	
	Others	9 (5.4)	
Malignant tumors	Primary intra osseous SCC	26 (16.8)	32 (19.6%)

### Distribution of OT cases by gender

Ameloblastoma was the predominant type of OT diagnosed among the male patients with male to female sex ratio of 1.27. Similarly, ameloblastoma and primary intra osseous SCC were the two most frequently diagnosed OTs among females. However, odontoma had equal distribution among both genders (Table 3).

**Table 3:** Distribution of odontogenic tumors by sex at St. Paul’s referral hospital, Addis Ababa, Ethiopia

Types of OTs	Gender		Total	Male to female ratio
	Male	Female		
Ameloblastoma	42	33	75	1.27
Primary intra osseous SCC	16	10	26	1.6
Odontogenic myxioma	10	4	14	2.5
Keratocystic OT	2	8	10	0.25
Odontogenic fibroma	4	6	10	0.67
Ameloblatic fibroma	6	3	9	2
Odontoma	3	3	6	1
Others	6	9	15	0.67
Total OTs cases	89	74	163	1.2

### Distribution of OT cases by age

The age of patients diagnosed with OT ranged from 8 to 80 years with a mean age of 34 years. 40 (24.5%) ameloblastoma cases were within the age group of 20–39 years. Primary IOSCC was seen predominantly among the age group of greater than or equals to 40 years. Odontogenic myxioma, odontogenic fibroma and odontoma were commonly seen among the age group of 20–39 years which accounted 7 (50%), 6 (75%) and 4 (66.7%), respectively. 5 (50%) of KCOT were diagnosed with the age range of 0–19 years old. Similar number of ameloblastic fibroma was seen in patients age group of 0–19 and 20–39. 11 (73.3%) of other type of OTs were seen in the age group of 20.39 years old (Table 4).

**Table 4:** Distribution of odontogenic tumors by age at St. Paul’s referral hospital, Addis Ababa, Ethiopia

Odontogenic tumors	Age (years)		
	0-19	20-39	> = 40
Ameloblastoma	17	40	18
Primary intra osseous SCC	0	7	19
Odontogenic myxioma	5	7	2
Keratocystic OT	5	3	2
Odontogenic fibroma	1	6	1
Ameloblatic fibroma	4	4	1
Odontoma	1	4	1
Others	1	11	3
Total OTs cases	34	82	47

### Distribution of OT case by location

OTs were located in different sites of maxillofacial areas. Mandible was the most frequently affected site constituting 126 (77.3%) of OT cases, while the maxilla was affected in 37 (22.7%) of OT cases. Of the different classes of OTs, ameloblastoma, primary IOSCC, odontogenic myxioma, and odontogenic fibroma were the most frequent forms OTs that were diagnosed in the mandible. Similarly, ameloblastoma, primary IOSCC and KCOT were the commonest OT types that affected maxilla (Table 5).

**Table 5:** Distribution of odontogenic tumors by site of occurrence at St. Paul’s referral hospital, Addis Ababa, Ethiopia

Odontogenic tumors	Maxilla	Mandible	Maxilla: Mandible
Ameloblastoma	8	67	0.18
Primary intra osseous SCC	8	18	0.44
Odontogenic myxioma	3	11	0.27
Keratocystic OT	6	4	1.5
Odontogenic fibroma	3	7	0.43
Ameloblatic fibroma	4	5	0.8
Odontoma	0	6	-
Others	5	10	0.5
Total OTs cases	37	126	0.3

### Surgical treatment and its complications

Out of the total 163 patients, 135 (82.8%) received surgical treatment while 28 (17.2%) were referred to other oncology centers. From the 135 patients who received primary treatment at SPRH, 80 (49.1%) of patients had segmental resection whilst 20 (12.3%) had marginal resection. In addition, enucleation and curettage were done for 35 (21.5%) of the treated patients (Table 6).

**Table 6:** Frequency distribution of surgical treatment and its complications of odontogenic tumors at St. Paul’s referral hospital, Addis Ababa, Ethiopia

Variables			Frequency (%)
Surgical treatment	Jaw Resection	Segmental	80 (49.1)
		Marginal	20 (12.3)
	Enucleation and curettage		35 (21.5)
	Referral to oncology center		28 (17.2)
Surgical Complication	Yes		116 (71.2)
	No		47 (28.8)
Type of surgical Complications	Facial disfigurement		66 (40.5)
	Malocclusion		56 (36.2)
	Numbness		13 (8.0)
	Recurrence		20 (12.3)
	Continuity defect		80 (49.1)
	Other		4 (2.5)

Facial disfiguration, malocclusion, numbness, recurrence, and continuity defects were the complications observed after surgical treatment in 66 (40.5%), 56 (36.2%), 13 (8.0%), 20 (12.3%) and 80 (49.1%) patients, respectively. Of note, out of these 135 patients who had surgical treatment, 47 of them had no any complication after surgery (Table 6).

Bivariate logistic regression analysis was conducted to assess the relationship between complications after surgery and different variables such as gender, age, place of residence, size and time of OT presentation. Importantly, complication after surgery had statistically significant relation with place of residence and tumor size. Living in the rural areas had more likely chance of having complication after surgery than living in the urban areas [Adjusted OR = 2.13, (95% CI: 0.98, 4.6)]. Similarly, as size of tumor increases the chances of having complication after surgery also increases [Adjusted OR = 4.24, (95% CI: 1.76, 10.21)]. However, gender, age, time of OT presentation were not statistically significant (*P* > 0.05) (Table 7).

**Table 7:** Bivariate analysis of complication after surgery for odontogenic tumor treatment and different factors at St. Paul’s referral hospital, Addis Ababa, Ethiopia

Variables		Complication after surgery		Crude OR	Adjusted OR	*P*-value
		Yes	No			
Gender	Female	55 (33.7%)	20 (12.3%)			0.97
	Male	61 (37.4%)	27 (16.6%)	0.82 (0.42,0.82)	1.02 (0.48,2.14)	
Age	0–19	27 (16.6%)	7 (4.3%)			0.16
	20–39	60 (36.8%)	22 (13.5%)	0.71 (0.27,1.85)	2.18 (0.74,6.42)	0.41
	> = 40	29 (17.8%)	18 (11.0%)	0.42 (0.15,1.16)	1.42 (0.61,3.28)	
Place of residence	Urban	53 (32.5%)	14 (8.6%)			0.05*
	Rural	63 (38.7%)	33 (20.2%)	1.98 (0.96,4.09)	2.13 (0.98,4.6)	
Tumor size	0–3 cm	23 (14.1%)	20 (12.3%)			0.186
	4–6 cm	28 (17.2%)	14 (8.6%)	1.74 (0.72,4.18)	1.86 (0.74,4.66)	0.001*
	> = 7 cm	65 (39.9%)	13 (8.0%)	4.35 (1.87,10.12)	4.24 (1.76,10.21)	
Time of OT presentation	<1 year	32 (19.6%)	19 (11.7%)			0.17
	> = 1 year	84 (51.5%)	28 (17.2%)	1.78 (0.88,3.63)	1.71 (0.8,3.67)	

## Discussion

Studies of OT among population are crucial for identification of the population group at risk, possible factors associated to OT development, and to develop more precisely differential diagnosis [13]. OTs studies are very scarce in Ethiopia. Therefore, we aimed at investigating the pattern of OTs in Ethiopia over a period of 8 years. In the current study, there were significant number of medical records that were excluded from the analysis. Of note, most of the complete records were for patients who had surgical treatment. In fact, most patients who had surgical treatment were with benign OT, which might in turn have led to the over representation of benign tumors over malignant ones. Therefore, it is important that the readers keep this in mind and interpret the date carefully.

Classes of OTs can either be benign or malignant. However, when considered globally, there is a considerable racial predilection for specific types of tumor. In this study, about 80.4% of the cases were benign OTs. This finding is slightly lower than reports from Nigeria (96.8%) [14] and (96.6%) [15], India (100%) [16], Turkey (93.2%) [12], and Brazil (94.5%) [13]. Some forms of benign OTs such as KCOT, odontoma do not exhibit clinical symptom. As a result, the chances of detecting them are really low unless otherwise patients had routine dental examination, which is unusual practice in Ethiopia. This might be a reason for low incidence of benign tumors in the current study. On the other hand, about 19.6% of this study cases were found to be malignant. This finding is higher than reports from Nigeria (3.2%) [14] and (3.4%) [15], Turkey (6.8%) [12] and Brazil (5.5%) [13]. The observed differences in the distribution of malignant OTs might be due to the geographical and cultural variation among the different study population. For instance, some of our study participants were using traditional medicines such as leaves, stems, and roots of some plants before coming to the hospital. Nevertheless, these traditional medications are not successful in curing maxillofacial related health problems. Thus, the use of traditional medicines among our study participants, in the context of maxillofacial problems, might increase the risk of transforming benign tumor into malignant tumor. Besides, most of our study patients sought dental and maxillofacial treatments at late stages of disease. This alone could contribute a lot to the transformation of benign OTs into malignant OTs [15, 17].

In the current study, ameloblastoma was the most frequent type of OTs accounting for about 46% of all OTs. This finding is consistent to reports from Nigeria [14, 15], India [16], Pakistan [18], Kenya [19], and Tanzania [20]. Primary IOSCC was found to be the second most common type of OTs (16%). This finding is lower than reports from Pakistan [2] and China [4], and higher than another report from China [3]. The observed variation in the distribution of primary IOSCC could probably be due to small sample size in the current study. Likewise, odontogenic myxoma was found to be the third most common type of OT in the current study which accounted for about 8.6%. This finding is consistent with a report from Nigeria [21]. Furthermore, the remaining OTs types such as KCOT, odontogenic fibroma, ameloblastic fibroma, odontoma, CCOT, cementoblastoma, malignant ameloblastoma, clear cell odontogenic tumor and gouts cell odontogenic tumor accounted for about 29.4% of the total OTs.

In the present study, most of OT cases were seen in males than females. This finding is in accordance with other reports from Pakistan [18], and Nigeria [9, 15]. In contrary, reports from Ceará, Brazil [7], Rio Grande do Norte, Brazil [10] and Mumbai, India [16] revealed a predominance of OTs among females than males. With respect to the distribution of specific OT types, the majority (56%) of ameloblastoma cases were seen among males. This finding is similar to different reports from Nigeria [9, 15, 21], India [16], and Argentina [22]. In addition, primary IOSCC was found to be the most frequent type of OTs in males 16 (61.5%), a finding consistent with two different reports from China [3, 4].

OTs were more frequent during the second and third decades of patients life. Previous reports from Ceará, Brazil [7], Rio Grande do Norte, Brazil [10], Pakistan [18], and Argentina [22] revealed similar results. In the present study, 40 (53.3%) ameloblastoma cases were identified within the age group of 20–39 years, a finding comparable to reports from Brazil [13], Nigeria [14, 15], India [16], and Pakistan [18]. Moreover, 19 (73.1%) of primary IOSCC were seen among patients with age of 40 years and older. This finding is in line with reports from China [3, 4].

About 77.3% of OTs were observed in the mandible, whereas the remaining 22.7% were located in the maxilla. This finding is similar to reports from Ceará, Brazil [7], Rio Grande do Norte, Brazil [10], Nigeria [14, 15], Mumbai, India [16], Pakistan [18], Kenya [19] Tanzania [20] and Buenos Aires, Argentina [22]. Mandible was found to be the most common site in about 89.3% of ameloblastoma cases in this study. This finding is comparable with reports from Nigeria [9, 15, 21], India [16], and Brazil [23, 24]. In addition, predilection of mandible was observed among the primary IOSCC, a finding consistent with reports from Pakistan [2] and China [3, 4].

Complication after surgical treatment of OTs is a commonly encountered phenomenon [14]. Unsurprisingly, about 71.2% of patients had complication after surgery. Of note, a patient could have more than one form of complications. About 12.3% of the cases had history of recurrence, a finding which is higher than a report from Nigeria [14]. In addition, the present study showed statistically significant association between two independent variables; place of residence and tumor size with complication after surgery. This shows that living in the rural areas lead to have higher chances of having complication after surgery than living in the urban areas. In addition, an increase in the size of tumor increased the chance of having complication after surgery. This association could be explained by the fact that dental services in general and maxillofacial services in particular are extremely scarce in Ethiopia, moreover in the rural parts. Thus, patients from the rural area would have a late presentation to dental and/or maxillofacial clinics. The late presentation of cases in turn could result in large sizes of OTs. As a consequence, necessitates a wide surgical procedures to be carried out for the treatment. Hence, patients will have a higher chance of having complications after surgery.

## Conclusions

The vast majority of patient’s medical records were incomplete and handled in appropriately. Therefore, the record archiving of the hospital needs to be improved, for instance, providing written data recording protocols, implementing a sustainable digital data archiving system, and providing continuous training and audit. Different patterns of OTs and their distribution among different age groups and genders were observed. Intriguingly, posterior mandible was the commonest site involved in OTs. Complication after surgery had a statistically significant association with tumor size and place of residency. Though the profession of dentistry is at the early stage of development in Ethiopia, the community has to bring behavioral changes in having habit of regular visit to dentists.
